# Clearance of Maedi-visna infection in a longitudinal study of naturally infected rams is associated with homozygosity for the *TMEM154* resistance allele

**DOI:** 10.1099/jmm.0.001506

**Published:** 2022-02-10

**Authors:** Scott Jones, Heather McKay, Laura Eden, Nicola Bollard, Stephen Dunham, Peers Davies, Rachael Tarlinton

**Affiliations:** ^1^​ School of Veterinary Medicine and Science, University of Nottingham, Sutton Bonington Campus, Loughborough LE12 5RD, UK; ^2^​ Department of Life Sciences, Imperial College London, London SW7 2AZ, UK; ^3^​ Three Valleys Veterinary, 107 Kesh Road, Irvinestown, Enniskillen BT94FX, UK; ^4^​ Bishopton Veterinary Group, Mill Farm, Studley Road, Ripon, North Yorkshire, HG4 2QR, UK; ^5^​ Department of Livestock and One Health, University of Liverpool, Liverpool, CH64 7TE, UK

**Keywords:** Maedi-visna, sheep, retrovirus, *TMEM154*, small ruminant lentivirus

## Abstract

Maedi-visna (MV) is a lentiviral disease of sheep responsible for severe production losses in affected flocks. There are no vaccination or treatment options with control reliant on test and cull strategies. The most common diagnostic methods used at present are combination ELISAs for Gag and Env proteins with virus variability making PCR diagnostics still largely an experimental tool. To assess variability in viral loads and diagnostic tests results, serology, DNA and RNA viral loads were measured in the blood of 12 naturally infected rams repeatedly blood sampled over 16 months. Six animals tested negative in one or more tests at one or more time points and would have been missed on screening programmes reliant on one test method or a single time point. In addition the one animal homozygous for the ‘K’ allele of the *TMEM154* E35K SNP maintained very low viral loads in all assays and apparently cleared infection to below detectable limits at the final time point it was sampled. This adds crucial data to the strong epidemiological evidence that this locus represents a genuine resistance marker for MV infection and is a strong candidate for selective breeding of sheep for resistance to disease.

## Introduction

Small ruminant lentiviruses (SRLVs) including Maedi-visna (MV) and Caprine Arthritis-Encephalitis (CAE) are lentiviral diseases of sheep and goats respectively that have a severe economic impact on infected flocks. SRLVs infect monocytes and remain dormant until these mature into macrophages, hence it has a very long latent period with clinical signs often not developing for several years. In sheep MV is primarily a respiratory disease with affected lungs greatly enlarged and displaying typical macrophage infiltrations, though encephalitis and mastitis are also clinical syndromes associated with virus infection [1]. SRLVs in common with other lentiviruses display extreme genetic hypervariability [2] which has meant that to date the development of effective vaccines has been unsuccessful; in some cases experimental vaccines have even enhanced infection [3]. Treatment of lentiviral disease with antiretroviral drugs in sheep is generally regarded as uneconomical and control programmes for the disease primarily rely on culling affected individuals; this can be very detrimental to affected flocks with infection rates of greater than 50 % in many flocks by the time clinical signs are noticed and the disease diagnosed [4].

Breed specific resistance to MV has long been recognised [5, 6] with increased interest in recent years in selecting for genetic resistance to the virus. Small deletions in the C-C chemokine receptor type 5 (*CCR5*) gene (known to convey resistance to human immunodeficiency virus) have been explored with equivocal results [7–10]. There is however increasingly strong evidence for an E/K (glutamic acid/lysine) substitution at position 35 in exon 2 of the transmembrane protein gene *TMEM154* being associated with resistance to MV in sheep. Mutations in *TMEM154* (function unknown) were first identified as potential MV resistance allelles in a genome wide association study of naturally infected ewes [11, 12]. Multiple large studies of naturally infected animals have demonstrated consistently lower infection rates in animals homozygous with the ‘K’ allele at this site [9, 12–16], with an estimated 2–3 fold lower risk of seropositivity for MV in affected flocks. Though resistance may be dependent on which virus strain animals are infected with, with some evidence that in animals homozygous for the ‘K’ allele or with larger deletions in this gene are more likely to carry particular virus subtypes [17, 18].

The hypervariablity of the virus and the low levels of viral DNA found in the blood during the latent stage has confounded attempts to develop PCR based diagnostics that will cover all strains. Current diagnostics are usually ELISAs based on a combination of Env and Gag proteins and are greater than 90 % specific and sensitive for detecting infected animals [19], they do however suffer from a long latent period between infection and sero-conversion meaning multiple screens of an affected flock are usually necessary to detect and remove all infected animals. Strain specific PCR tests can detect infection earlier than serology tests but to date remain only in experimental use [19–22].

There are few longitudinal studies of Maedi-visna virus (MVV) infected animals outside of short term experimental settings [23–26]. Most studies following the same infected animals for extended periods of time pre-date the diagnostics in use today [27, 28] meaning we have little idea what the variability in antibody and viral titre in affected animals is and how this may potentially confound screening programmes for the disease. To assess variability in viral load and diagnostic test results this study followed a group of twelve naturally infected rams for 16 months, testing them at two or three time points with commercially available ELISA serology tests for MV and strain specific qPCRs for DNA and RNA viral loads in the blood. The animals displayed no consistent patterns in viral load with the three tests not correlating well with each other. Six animals tested negative at one time point for one or more tests, highlighting why repeated testing with multiple tests may be necessary to identify all infected animals.

Animals were additionally genotyped for *TMEM154* allelles. One animal apparently cleared the virus to below detectable limits in all tests at its final sampling point. This animal was the only one in the study homozygous for the K allele at E35K *TMEM154*, raising the possibility that in addition to providing resistance to initial infection animals with this allele may be better able to control virus infection.

## Methods

Five Aberfield and seven Abermax MVV seropositive rams (12 in total) were included in this study. Rams were identified as being MVV positive during routine testing (Elitest ELISA Hyphen Biomed) as part of a MV surveillance scheme (Scotland’s Rural University College premium sheep health and goat scheme) after which they were acquired by the University of Nottingham in 2015 (at approximately 18 months of age). Animals were held at pasture with available shelter and supplementary rations. Five animals were culled for flock management purposes in June and July 2016 with the seven surviving rams euthanased in October 2016 (Table 1).

**Table 1.** Animals used in this study

Ram ID	Breed	Date of death
Trial rams (kept for the full time)		
2	Aberfield	28/10/16
3	Aberfield	28/10/16
4	Abermax	28/10/16
6	Aberfield	28/10/16
7	Abermax	28/10/16
9	Aberfield	28/10/16
10	Aberfield	28/10/16
Rams euthanased		
22	Abermax	23/05/16
24	Abermax	02/06/16
25	Abermax	02/06/16
26	Abermax	20/06/16
27	Abermax	20/06/16

For detection of MVV, blood was collected from seropositive rams in April 2015, December 2015 and in October 2016 for the surviving rams. Blood was collected in 10 ml vacutainer blood tubes taken from the jugular vein. For separation of sera from blood, samples were left at room temperature overnight to allow for clotting after which sera was removed via pipetting. Sera and blood clots were stored at −20 °C.

Sera was tested for the presence of SRLVs specific antibodies using the MVV/CAEV p28 Antibody Screening Test ELISA (IDEXX, Netherlands) following the manufacturer’s recommended protocol. Samples were run in triplicate.

DNA was extracted from blood clots or tissue samples using the Nucleospin Tissue kit (Macherey-Nagel, Hoerdt France) using the protocol for extraction of genomic and viral DNA from blood samples or tissues. A minor change of the addition of 200 ul PBS and a sterile 5 mm steel bead to the blood clots for homogenization by Retsch MM300 bead mill (Qiagen) at a frequency of 25 s^−1^ for 2 min was made. DNA was stored at −20 °C until use.

RNA was extracted from sera isolated using the QIAamp Viral RNA Mini Kit (Qiagen, Manchester, UK) as per manufacturers protocol.

DNA and RNA quantification was performed using the NanoDrop 8000 Spectrophotometer (Thermo Fisher UK).

Reverse transcription of RNA was carried out using random hexamer primers (Thermo Scientific) and M-MLV (Promega) or AMV (NEB) reverse transcriptase (RT) as per manufacturer's protocol with cDNA stored at −20 °C before use. For detection of MVV sequences from extracted nucleic acids, a Sybr green based qPCR procedure was carried out with primers targeting the *pol* gene specific to the strain of MVV isolated from these animals (publication in preparation) Pol1F (AGGGGATGCATACTTTACTATACCA) and Pol1R (TCTTGTGCATGGCCCTAAAT). Reaction mixtures consisted of 1 × qPCRBIO SyGreen Mix Lo-ROX master mix (PCR Biosystems), 0.04 µM forward and reverse primers (Sigma-Aldrich) and 1 µl of test DNA or standard in a total volume of 20 µl. Reaction conditions consisted of a starting incubation of 95 °C for 15 min followed by 45 cycles of 95 °C for 5 s, 60 °C for 30 s and 72 °C for 10 s. A melt cycle was carried out at reaction end ranging from 65–95 °C. Reactions were carried out within a CFX Connect Real-Time PCR Detection System (Biorad Laboratories). A 1 : 10 standard dilution series of a synthesised PCR product positive control was run with each plate. Reactions were run in duplicate.

*TMEM154* genotyping was carried out by PCR and Sanger sequencing of exon two using primers from [12]: 86 824 (TCCATTTCCTTTACCTAAAAGT) and 86 826 (ACTGGCCCAAATTACATAAG). PCR was carried out with 1 µl of DNA extracted from the lung tissue of MVV seropositive rams, used in a reaction mixture of 25 µl. Each reaction contained five units of *Taq* DNA Polymerase, 1 × standard *Taq* (Mg-free) reaction buffer (NEB), 3 mM magnesium chloride (MgCl2) (NEB), 0.04 pmol of forward and reverse primers and 0.4 mM deoxynucleotide (dNTP) solution mix (Thermo Scientific). Standard PCR cycling conditions consisted of an initial denaturation phase of 95 °C for 5 min followed by 45 cycles of 95 °C, 56/60 and 68 °C, each for 15–60 s. Reactions were carried out within a Thermal cycler Life ECO (Bioer Technology). Successful amplification was determined by agarose gel electrophoresis of PCR products. Products were purified for sequencing using the Nucleospin Gel and PCR clean up kit (Machery Nagel) following the manufacturers protocol for PCR clean up. Sanger sequencing was carried out by Source Bioscience (Nottingham). Sequence analysis was carried out using BioEdit v7.2 and CLC Sequence Viewer software v8.0 (Qiagen).

## Results

**Fig. 1.**
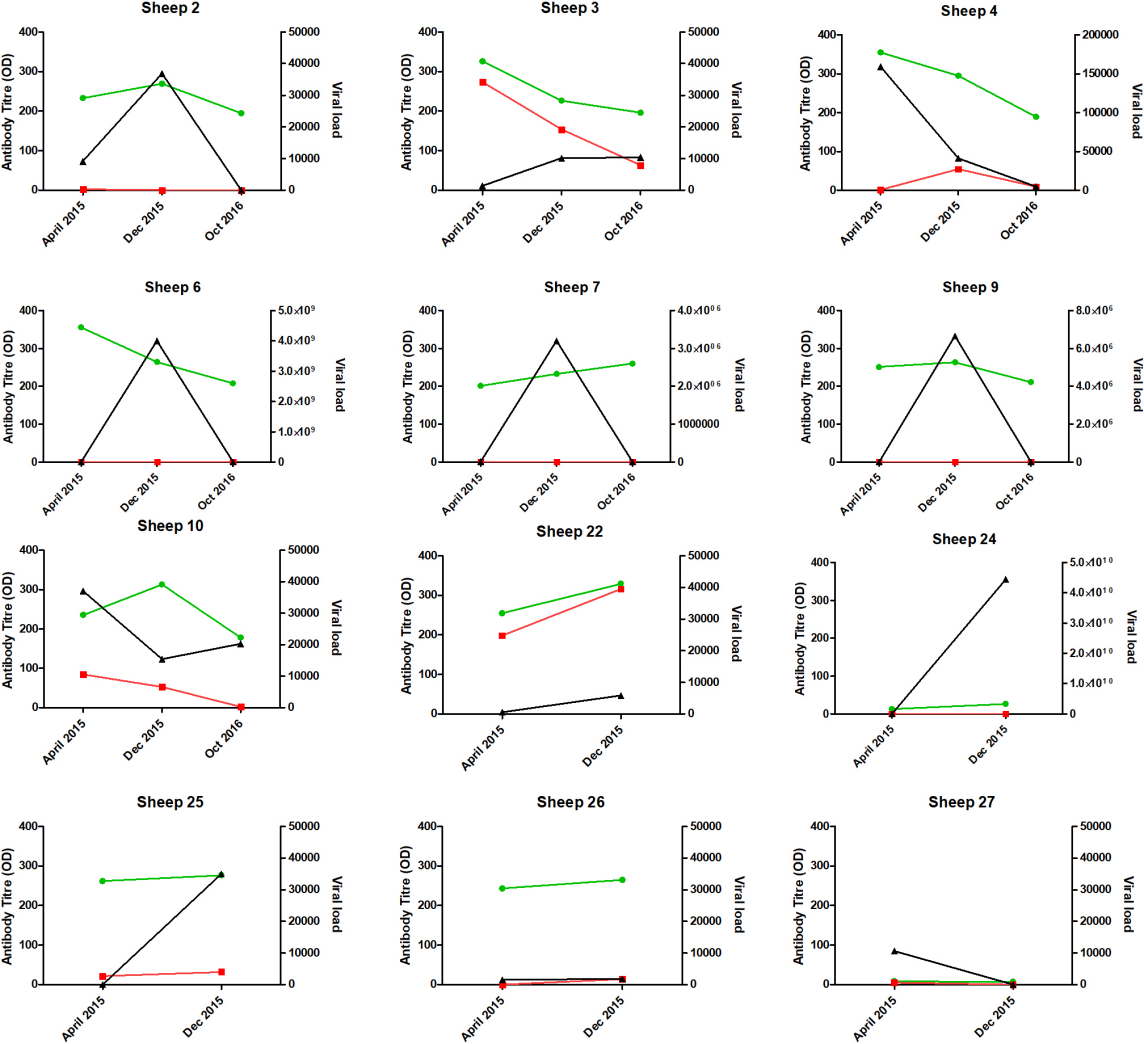
Maedi-visna testing results per ram. Green line: antibody titres optical density (left axis), red line: viral DNA load ng^−1^ DNA (right axis), black line: viral RNA load ul^−1^ serum (right axis), x axis: date of sample collection, sheep 22–27 have only two data points.

There was no clear pattern of variation in ELISA, DNA or RNA loads in the studied animals (Fig. 1, Table 2). Antibody titres, DNA and RNA loads did not rise or fall consistently with each other or consistently increase or decrease over time. DNA loads in all animals were consistently below 40 000 copies per ng DNA, whereas RNA loads varied much more dramatically with one animal reaching titres of 4.44×10^10^ copies per µl serum (Table 2). Six different animals tested negative with different assays at one or more time points in the study.

**Table 2.** Testing results, ELISA results are optical density as per IDEXX test kit, DNA viral load ng^−1^ DNA, RNA viral load ul^−1^ serum. Negative results (zero for RNA or DNA or below assay cut off for ELISA) are shown in bold *TMEM154* genotype at amino acid position 35

Ram	April 2015			Dec 2015			October 2016			TMEM154 genotype at position 35 exon 2
	ELISA	DNA	RNA	ELISA	DNA	RNA	ELISA	DNA	RNA	
2	233	315	9150	269	**0**	36 900	195	**0**	**0**	E/E
3	326	34 200	1400	227	19 200	10 200	196	7910	10 400	E/K
4	355	743	159 000	295	27 400	41 180	189	4700	4620	E/K
6	355	1940	1180	264	523	4000000000	207	**0**	10 500	E/K
7	201	417	10 900	233	825	3 200 000	260	**0**	2170	E/K
9	251	**0**	1180	263	1590	6 650 000	211	227	7100	E/E
10	235	10 600	37 000	313	6590	15 400	178	293	20 300	E/E
22	255	24 800	607	329	39 500	5840	–	–	–	E/K
24	**13**	3900	46 200	**27**	8860	44400000000	–	–	–	E/K
25	262	2740	**0**	277	4020	35 000	–	–	–	E/K
26	243	881	1560	265	1730	1800	–	–	–	E/K
27	**9**	693	10 600	**7**	**0**	**0**	–	–	–	K/K

Genotyping of exon 2 of the *TMEM154* gene demonstrated 8/12 animals heterozygous for the wild-type and resistant alleles at amino acid 35 (E/K), 3/12 animals homozygous for the wild-type allele (E/E) and one animal homozygous for the resistant allele (K/K) (Table 2). Sequences of the *TMEM154* exon 2 (that differ from the wild-type) have been submitted to European nucleotide archive with the accession numbers (OU642616-OU642637). The animal homozygous for the resistant allele demonstrated ELISA titres consistently below the cut off for the assay and tested negative in all three assays at the last time point before its death. There was no clear change in titres for animals homozygous for the wild-type at this allele.

## Discussion

The results of this study demonstrate some of the confounding factors with current MV diagnostics. All studied animals had a positive serology test for MV before the study and all tested positive for the virus in PCR tests at least once during the course of this study. However six animals tested negative in one or more tests (serology or PCR) at least once. Two animals tested negative on serology and would have been missed on a single screening of the flock conducted at this time. Screening and control strategies for MV rely on multiple serology screens of flocks at least 6 months apart as in the UK's premium sheep health and goat schemes [29]. Multiple studies have demonstrated that the addition of PCR based blood testing to serology screening improves the rate of detection of infected animals [20, 30, 31], though the uptake of PCR based diagnostics for MV has been limited both by cost and the variability of the virus (making it difficult to design a PCR based test that will detect all strains) [32]. This study supports the necessity of multiple serology screening points to detect all infected animals. Although the viral RNA loads are quite variable, we consider them likely genuine rather than as a result of sequence variability as this should also have been evident in the DNA loads, which while higher than previous studies are within a much more limited range [23, 33, 34]. This may well reflect differences between proviral copies integrated into macrophage genomes and infectious viral RNA particles being released from these cells.

The other major finding of this study is that one animal homozygous for the ‘K’ allele of *TMEM154* at position 35 had a consistently low viral load in all three tests and apparently cleared the virus to below detectable limits in all assays by the final time point in the study. Extensive genetic and virus prevalence screening of MV infected flocks in multiple countries has demonstrated that sheep homozygous for the resistant allele of *TMEM154* are less likely to test positive for the virus and have lower viral loads [8, 9, 11–13, 15, 16]. While only a single animal, this study adds a crucial piece of evidence suggesting that sheep with this allele are also able to clear a previous infection to below detectable levels. Although, we cannot exclude the possibility of the animal having a very low level latent infection below the detection limits of the assay, the study still adds more evidence to the body of data that homozygosity for K at *TMEM154* E35K represents a genuine genetic resistance marker for MV. Genomic selection for for genetic resistance to disease has been used successfully to virtually eradicate other infectious diseases such as scrapie in UK sheep flocks [35]. This is an attractive option for disease control in commercial sheep flocks, although possible adverse impacts on production traits should be studied for this genetic selection in follow-up studies.
